# Psoriasis and Risk of Uveitis: A Systematic Review and Meta-Analysis

**DOI:** 10.1155/2020/9308341

**Published:** 2020-07-15

**Authors:** Chayada Chaiyabutr, Patompong Ungprasert, Narumol Silpa-archa, Chanisada Wongpraparut, Leena Chularojanamontri

**Affiliations:** ^1^Department of Dermatology, Faculty of Medicine Siriraj Hospital, Mahidol University, Bangkok, Thailand; ^2^Clinical Epidemiology Unit, Department of Research and Development, Faculty of Medicine Siriraj Hospital, Mahidol University, Bangkok, Thailand

## Abstract

**Background:**

Uveitis is a known ophthalmologic manifestation of seronegative spondyloarthropathy, including psoriatic arthritis. However, the data is less clear among patients with psoriasis due to the limited number of published studies.

**Aims:**

To investigate whether the risk of incident and prevalent uveitis is elevated among patients with psoriasis using systematic review and meta-analysis technique.

**Methods:**

The MEDLINE and EMBASE databases were searched from their inception to May 2019. Eligible studies must have included a psoriasis group and a nonpsoriasis group. Eligible studies must also have investigated for prevalent or incident uveitis, and the magnitude of difference between the study groups must have been reported. Pooled risk ratio and 95% confidence interval (CI) were calculated using random-effect generic inverse variance methods.

**Results:**

Of 7,107 potentially eligible articles from the EMBASE and MEDLINE databases, 7 studies were included in the meta-analysis. Two of those studies compared the incidence, and 5 studies compared the prevalence of uveitis between the psoriasis and nonpsoriasis groups. For incident uveitis, a total of 5,865,801 patients (222,083 with psoriasis and 5,643,718 without psoriasis) were analyzed. For prevalent uveitis, a total of 1,343,436 patients (37,891 with psoriasis and 1,305,545 without psoriasis) were studied. The risk of incident uveitis was significantly higher among patients with psoriasis with a pooled risk ratio of 1.23 (95% CI: 1.05-1.45, *I*^2^ = 55%). The risk of prevalent uveitis was also significantly higher among patients with psoriasis with a pooled risk ratio of 1.97 (95% CI: 1.68-2.31, *I*^2^ = 0%).

**Conclusions:**

The results of this study revealed significantly increased risk of both prevalent and incident uveitis among patients with psoriasis.

## 1. Introduction

Psoriasis is a common chronic inflammatory skin disease with a reported prevalence that ranges from 0.5% to 11.4% depending on geographic region [1]. Its pathogenesis is related to autoimmunity and systemic inflammation, which can result in multiorgan involvement and increased risk of comorbidities. Ocular involvement is being increasingly recognized as a complication of psoriasis [2–4]. The possible mechanisms of ocular involvement include direct involvement of psoriatic skin lesion to the epithelial component of the eye, shared genetic predisposition, and adverse effect of psoriasis treatment, such as excessive use of topical corticosteroids and prolonged course of psoralen-UVA (PUVA) photochemotherapy [2].

Uveitis is characterized by inflammation of the uveal tract, which includes the iris, ciliary body, choroid tissue, and adjacent structures [5]. The reported global prevalence of uveitis ranged from 38 to 714 cases per 100,000 individuals. If left untreated, uveitis can lead to several complications that can lead to permanent visual impairment [6]. Uveitis is a known ophthalmologic manifestation of several seronegative spondyloarthropathies, such as ankylosing spondylitis, reactive arthritis, and psoriatic arthritis. It is also being increasingly reported in psoriasis [7]. However, the results of studies of uveitis in psoriasis are fairly heterogeneous, and some studies are just descriptive studies without comparative analysis [3, 8–11]. The aim of this study was to better understand and characterize the association between psoriasis and uveitis by identifying all related studies and systematically summarizing their results.

## 2. Materials and Methods

### 2.1. Search Strategy

Two investigators (C.C. and L.C.) independently searched published studies indexed in the MEDLINE and EMBASE databases from inception to May 2019. The search strategy included terms for “psoriasis” and “eye disease,” as described in Table S1. Eligible studies must include (i) a cohort of patient with adult psoriasis (cases) and a cohort of patients without psoriasis (comparators) and (ii) reported the difference of prevalent or incident uveitis between the two study groups. The reported difference could be in the form of odds ratio (OR), relative risk (RR), hazard ratio (HR), or standardized incidence ratio (SI) with 95% confidence interval (CI). Alternatively, if those ratios were not reported, sufficient raw data to calculate them was considered acceptable. Non-full-text articles were excluded.

The title and abstract of the retrieved articles were independently reviewed by two investigators (C.C. and L.C.). Articles that obviously did not fulfill the inclusion criteria were screened out during this round. Full-text articles among the remaining potentially eligible articles were then independently reviewed by the same two investigators for final determination of their inclusion eligibility. Any disagreement in the determination of reviewed studies was resolved by discussion and consensus between the two investigators. Quality assessment of the included studies was performed using the Newcastle–Ottawa quality assessment scale. This tool is specifically designed for assessment of quality of nonrandomized study, such as case-control and cohort study. It assesses the quality based on three aspects, including the selection of case and control, the comparability between the groups, and the ascertainment of the outcome of interest for cohort study and exposure of interest for case-control study [12]. Study with score ≥ 7 is generally considered as a high-quality study [13].

### 2.2. Data Extraction

The following data were extracted using a standardized data collection form: first author's name, title of the study, journal name, year of publication, year when the study was conducted, country where the study was conducted, study design, method used to diagnose psoriasis and uveitis, recruitment of cases and comparators, number and baseline characteristics of cases and comparators, follow-up duration, variables that were adjusted in multivariate analysis, and adjusted effect estimates with corresponding 95% CIs. Data extraction was independently performed by C.C. and L.C. to minimize any errors.

### 2.3. Statistical Analysis

Statistical analysis was performed using Review Manager 5.3 software from the Cochrane Collaboration (London, UK). Result of each study was combined to calculate the pooled effect using the generic inverse variance method of DerSimonian and Laird [14]. This method gives higher weight for the pooled analysis to study with higher precision. The weight for each study is in reverse to its variance as study with higher variance is study with less precision. The random-effect model was used in this analysis because the assumption of fixed-effect model that every study should give rise to the same result is generally not true, particularly in observational studies. Cochran's *Q* test and *I*^2^ statistic were used to assess for between-study statistical heterogeneity [15]. This *I*^2^ statistic was used to quantify the proportion of the total variation across studies that was from heterogeneity rather than chance. A value of *I*^2^ of 0–25% represents insignificant heterogeneity, 26–50% low heterogeneity, 51–75% moderate heterogeneity, and>75% high heterogeneity. Visualization by funnel plot was used for determination of publication bias if there were enough eligible studies to create the plot. Publication bias would be suspected if the funnel plot is asymmetric.

## 3. Results

The search strategy identified 7,107 potentially eligible articles from the EMBASE and MEDLINE databases. After exclusion of 671 duplicate articles, the titles and abstracts of the remaining 6,436 articles were reviewed. That review yielded 50 studies for full-text review. Forty-three of those studies were excluded for the following reasons: descriptive study (*n* = 16), review or commentary (*n* = 14), did not report an outcome of interest (*n* = 6), no event of uveitis in the study (*n* = 4), and not published in English language (*n* = 3). The remaining 7 seven studies were included in the meta-analysis.

**Figure 1:**
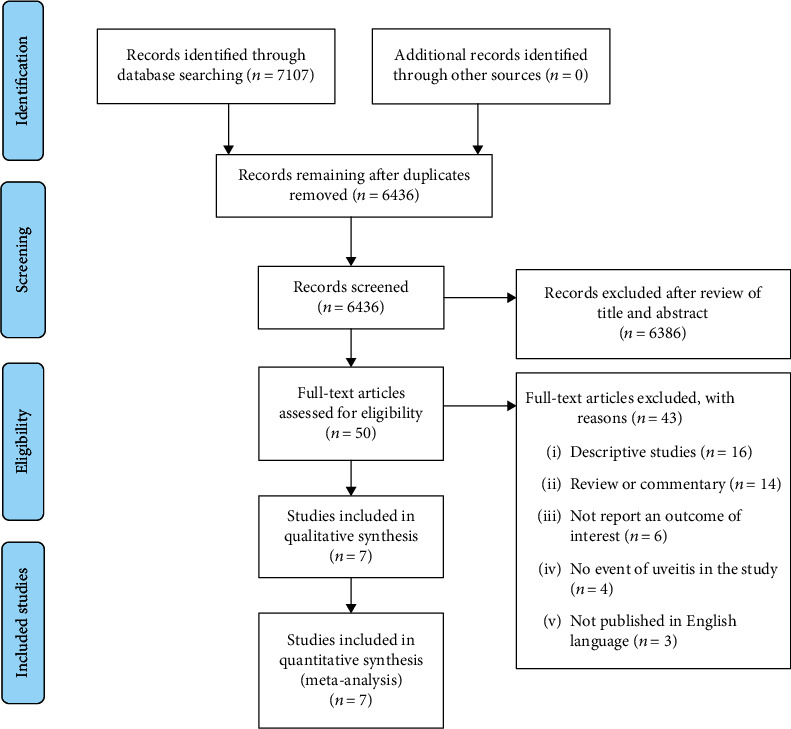
Flow chart describing the literature review and selection process.

Of those, 2 studies [16, 17] compared the incidence, and 5 studies [4, 18–21] compared the prevalence of uveitis between the psoriasis and nonpsoriasis groups. A flowchart describing the literature review and selection process is shown in Figure 1. The detail of psoriasis and nonpsoriasis group from each study, characteristics of the included incidence, and prevalence studies and quality assessment are shown in Tables 1 and 2, respectively.

The incidence of uveitis ranged from 2.9 per 10,000 person-years in the study by Egeberg et al. [17] to 10.4 per 10,000 person-years in the study by Chi et al. [16]. The prevalence of uveitis ranged from 0.4% in the study by Radtke et al. [20] to 14.3% in the study by Türkcü et al. [21].

### 3.1. Risk of Incident Uveitis

**Figure 2:**
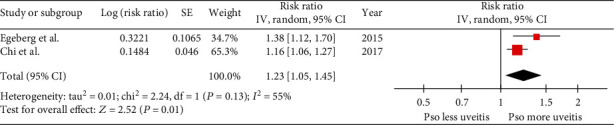
Forest plot of risk of incident uveitis.

A total of 222,083 patients with psoriasis and 5,643,718 individuals without psoriasis were included in the incident uveitis analysis. The risk of incident uveitis was significantly higher among patients with psoriasis than among those without psoriasis with a pooled risk ratio of 1.23 (95% CI: 1.05-1.45). The statistical heterogeneity was moderate (*I*^2^ = 55%) (Figure 2). The quality of both studies was good. Evaluation for publication bias using funnel plot was not performed due to the small number of included studies.

### 3.2. Risk of Prevalent Uveitis

**Figure 3:**
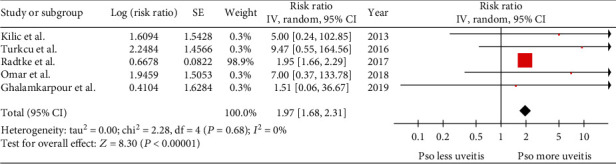
Forest plot of risk of prevalent uveitis.

**Figure 4:**
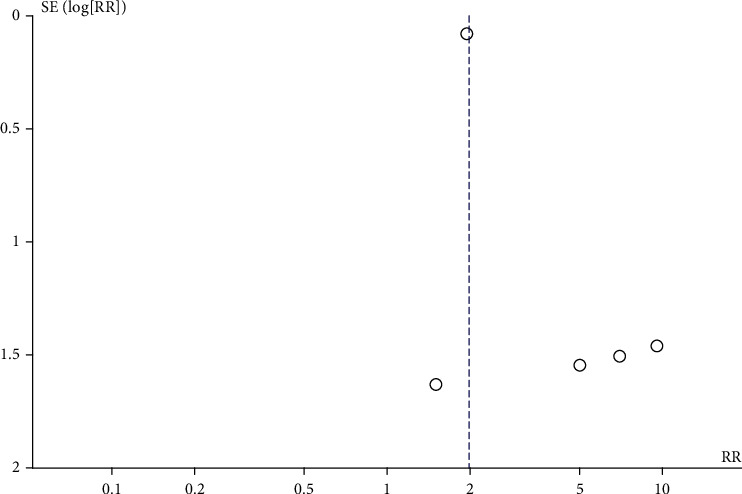
Funnel plot of prevalent uveitis.

A total of 37,891 patients with psoriasis and 1,305,545 individuals without psoriasis were included in the prevalent uveitis analysis. The risk of prevalent uveitis was also significantly higher among patients with psoriasis than among nonpsoriasis subjects with a pooled risk ratio of 1.97 (95% CI: 1.68-2.31). The quality of the included studies was fair to good. The heterogeneity analysis revealed no statistical heterogeneity (*I*^2^ = 0%) (Figure 3). Funnel plot was relatively symmetric and was not suggestive of the presence of publication bias (Figure 4).

**Figure 5:**
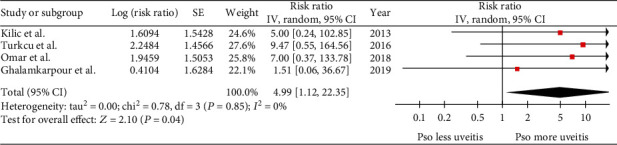
Sensitivity analysis of risk of prevalent uveitis.

Sensitivity analysis was conducted to explore whether the pooled result would be significantly different if the dominant study was not included. After excluding the study by Radtke et al. [20], we found that the risk of prevalent uveitis remained significantly higher among patients with psoriasis than individuals without psoriasis. The new pooled risk ratio was increased to 4.99 (95% CI: 1.12-22.35; *I*^2^ = 0%) (Figure 5).

## 4. Discussion

Uveitis is a known ophthalmologic manifestation of several systemic diseases, including seronegative spondyloarthropathy [7]. Acute unilateral anterior uveitis is the classic and well-recognized inflammatory eye disease in patients with ankylosing spondylitis, but all of the diseases in the seronegative spondyloarthropathy group could manifest with uveitis, including psoriatic arthritis [22]. However, the data is less clear among patients with psoriasis due to the limited number of studies addressing this issue. Accordingly, the present systematic review and meta-analysis combined all of the available data and found both incident and prevalent uveitis to be significantly more common among patients with psoriasis than among general population. For a sensitivity analysis, the study by Radtke et al. [20] was excluded from the full analysis because this study received almost 99% of weight in the pooled analysis as it was conducted using a very large administrative database. Interestingly, even after excluding this study, the pooled risk ratio was increased from 1.97 to 4.99. This finding supports that patients with psoriasis have a higher risk of prevalent uveitis than individuals without psoriasis.

There are few possible explanations for the observed increased risk of uveitis among psoriasis patients. The first explanation is related to the increased systematic inflammatory burden among patients with psoriasis that may also trigger ocular inflammation. In fact, several cytokines that are known to be key players in the pathogenesis of psoriasis, such as tumor necrosis factor- (TNF-) alpha, interleukin- (IL-) 2, IL-6, and IL-17, are also found to be at an increased concentration in the aqueous humor of patients with uveitis [23, 24]. Second, both diseases may share some genetic predisposition that could ultimately lead to autoimmunity in both the eye and the skin. One widely known example is HLA-B27, which is known to be associated with both psoriatic arthritis and uveitis [25, 26].

There are case reports of patients with psoriasis and other immune-mediated inflammatory diseases who developed uveitis after exposure to TNF-alpha inhibitor [27]. It is possible that they are true cases of TNF-alpha inhibitor-induced uveitis, and thus, use of TNF-alpha inhibitor could be one of the explanations for the increased prevalence/incidence of uveitis among patients with psoriasis. However, it is more likely that those patients developed uveitis because of their underlying disease (such as seronegative spondyloarthropathies) rather than as a complication of TNF-alpha inhibitor since TNF-alpha inhibitor has been extensively shown to be beneficial for mostly all types of uveitis [28].

## 5. Limitations

This study has some limitations that should be acknowledged. First, most included studies relied on diagnostic codes to make the diagnosis of psoriasis and uveitis, which may have limited the diagnostic accuracy for both conditions. This is of particular concern for uveitis as other inflammatory orbital diseases may be erroneously coded as uveitis. Additionally, traumatic anterior uveitis, which is not an autoimmune-related uveitis, could be coded under the same diagnostic codes for uveitis. This also limits the ability to further characterize subtype of uveitis. Second, moderate heterogeneity was observed in the meta-analysis of incident uveitis, which suggests that the included studies may have been somewhat too different to combine. Third, the quality of some of the included studies was low as reflected by the low Newcastle–Ottawa scores. The last point is that some of psoriasis patients in our cohort may have psoriasis in conjunction with psoriatic arthritis. Therefore, this result may not totally reflect the risk of uveitis in patients who have only skin psoriasis.

## 6. Conclusion

The results of this study revealed significantly increased risk of both prevalent and incident uveitis among patients with psoriasis. Dermatologists should regularly monitor these patients for eye symptoms for prompt recognition and treatment to prevent irreversible ocular complications.

## Figures and Tables

**Table 1:** Characteristics of included incident studies.

	Egeberg et al. [17]	Chi et al. [16]
Country of origin	Denmark	Taiwan
Study design	Cohort study	Cohort study
Year of publication	2015	2017
Cases	Cases of adults (18 years or older) with psoriasis were identified from the Danish National Patient Registry during 1 January 1997 to 31 December 2011. This registry covers all citizens of Denmark. Diagnosis of psoriasis was made based on the presence of diagnostic codes of psoriasis (ICD-8 code of 696.10 or 696.19 or ICD-10 code of L40, which are codes for psoriasis in general) or the presence of at least two prescriptions of topical vitamin D derivatives in the absence of ICD-8 code of 696.09 or ICD-10 code of M070-M073, which are the codes for psoriatic arthritis. Cases with diagnosis of uveitis prior to index date were not included.	Cases of psoriasis were identified from the National Health Insurance Research Database during 2000 to 2011. This registry covers 99% of the citizens of Taiwan. Diagnosis of psoriasis was made based on the presence of diagnostic codes of psoriasis (ICD-9-CM code of 696, 696.1, or 696.8, which are codes for psoriasis in general), in the absence of ICD-9-CM code of 696.0, which is the code for psoriatic arthritis. Cases with diagnosis of uveitis prior to index date were not included.
Comparators	Comparators were the rest of patients in the database who did not carry the diagnostic codes of psoriasis/psoriatic arthritis. Comparators with diagnosis of uveitis prior to index date were not included.	Gender- and age-matched comparators without a diagnosis code for psoriasis were randomly selected from the 2005 Longitudinal Health Insurance Database, which is a random subset of one million enrollees in the National Health Insurance Research Database for the year 2005. Comparators with diagnosis of uveitis prior to index date were not included.
Diagnosis of incident uveitis	The presence of the first diagnosis code for uveitis in the database after the index date.	The presence of the first diagnosis code for uveitis in the database after the index date (at least twice for outpatient visit or once for hospital admission).
Number of subjects (cases/comparators)	74,129/5,434,749	147,954/147,954
Percentage of female gender (cases/comparators)	50.6/51.6	41.2/41.2
Mean age in years (cases/comparators)	40.7/43.3	44.4/44.4
Follow-up	Until 31 December 2011, a diagnosis of end point, migration, or death from any causes.	Until incident uveitis, 31 December 2012, or withdrawal from the National Health Insurance program.
Confounder assessed in the multivariate analysis	Age, gender, socioeconomic status, and comorbidities	Age, gender, hypertension, dyslipidaemia, and diabetes
Quality assessment	Selection: 4 stars Comparability: 1 star Outcome: 3 stars	Selection: 4 stars Comparability: 2 stars Outcome: 3 stars

**Table 2:** Characteristics of included prevalent studies.

	Kilic et al. [18]	Radtke et al. [20]	Türkcü et al. [21]	Omar and Helaly [19]	Ghalamkarpour et al. [4]
Country of origin	Turkey	Germany	Turkey	Egypt	Iran
Study design	Cohort study	Cohort study	Cohort study	Cohort study	Cohort study
Year of publication	2013	2016	2016	2018	2019
Cases	Cases of adults (18 or older) with psoriasis were consecutively recruited from outpatient clinic of the study hospital. Diagnosis of psoriasis was made based on dermatological and histopathological evaluation. Exclusion criteria included the presence of other systemic diseases likely to have ocular involvement, history of eye surgery, eye trauma, primary eye diseases, and wearing contact lenses.	Cases of adults (18 years or older) with psoriasis were identified from the database of a German nationwide statutory health insurance entity named Gmuender Ersatzkasse that covered 1.6 million individuals. The database of the year 2009 was used. Diagnosis of psoriasis was made based on the presence of diagnostic codes of psoriasis.	Cases with psoriasis who were followed by the dermatology clinic of the study hospital were enrolled. Exclusion criteria included prior ocular surgery, retinal or choroidal pathology, high myopia and hypermetropia, best-corrected visual acuity less than 20/25, systemic disease (hypertension, diabetes mellitus, cerebrovascular disease, peripheral vascular disease, pregnancy, or lactation), and history of systemic treatment for psoriasis.	Cases with psoriasis were recruited from the outpatient clinic at the Faculty of Medicine, Alexandria University, Alexandria, Egypt Exclusion criteria included inflammatory connective tissue diseases, diabetes, nephropathy, lung and heart disease, gastroenterological disease/inflammatory bowel disease, neurological disease, neoplasia, metabolic bone disease, skin diseases other than psoriasis, infections, hematological disease, liver disease, previous ocular surgery, active eye infection, or active ocular allergy.	Cases of adults (18 years or older) with psoriasis were recruited from two dermatology centers from September 2014 to January 2017. Exclusion criteria included diabetes mellitus, hypertension, hyperlipidemia, thyroid disorders, hepatic or renal insufficiency, rheumatoid arthritis, gout, collagen vascular diseases, atopic dermatitis, rosacea, other chronic inflammatory diseases, pregnant or lactating women, smokers, contact lens wearers, use of anticholinergics, history of eye trauma or surgery, optic neuritis, glaucoma, radiotherapy, malignancy or chronic infection of the lacrimal glands, human immunodeficiency virus, and hepatitis B or C virus infections.
Comparators	Comparators without psoriasis and with healthy first-degree relatives were consecutively recruited from the same center.	Comparators were the rest of patients in the database who did not carry the diagnostic codes of psoriasis	Comparators were individuals without psoriasis who were admitted to the outpatient Clinic of Ophthalmology for minor refractive errors. They were gender- and age-matched to cases.	Comparators were individuals without psoriasis who were seen at the same clinic. They were gender- and age-matched to cases.	Comparators without psoriasis were recruited from the same centers.
Diagnosis of prevalent uveitis	Based on examination by ophthalmologists.	The presence of the first diagnosis code for uveitis in the database.	Based on examination by ophthalmologists.	Based on examination by ophthalmologists.	Based on examination by ophthalmologists.
Number of subjects (cases/comparators)	100/100	37,456/1,305,215	35/30	100/100	200/100
Percentage of female gender (cases/comparators)	52.0/52.0	Not available	54.3/50.0	38.0/38.0	46.0/46.0
Mean age in years (cases/comparators)	40.3/40.5	Not available	35.2/34.2	50.7/51.1	38.0/38.2
Confounder assessed in multivariate analysis	None	None	None	None	None
Quality assessment (Newcastle–Ottawa scale)	Selection: 3 stars Comparability: 1 star Exposure: 3 stars	Selection: 3 stars Comparability: 0 star Exposure: 3 stars	Selection: 2 stars Comparability: 1 star Exposure: 3 stars	Selection: 2 stars Comparability: 1 star Exposure: 3 stars	Selection: 2 stars Comparability: 1 star Exposure: 3 stars

## Supplementary Materials

Supplementary Materials Table S1: database search strategy.
