# Supplementary material for: Psoriasis and Risk of Uveitis: A Systematic Review and Meta-Analysis
Source: Biomed Res Int. 2020 Jul 15;2020:9308341. doi: 10.1155/2020/9308341 (PMC7381949; doi:10.1155/2020/9308341)
Supplement: Supplementary Materials — Table S1: database search strategy. [file 9308341.f1.docx]

**Table S1.** Database search strategy

| **Search strategy** |
| --- |
| **Database** |
| **MEDLINE**   1. Psoriasis.mp. OR exp Psoriasis/ 2. Eye disease.mp. OR exp Eye disease/ 3. Uveitis.mp. OR exp Uveitis/ 4. Iritis.mp. OR exp Iritis/ 5. Iridocyclitis.mp. OR exp Iridocylitis/ 6. Choroiditis.mp. OR exp Choroiditis/ 7. 2 OR 3 OR 4 OR 5 OR 6 8. 1 AND 7 |
| **EMBASE**   1. ‘psoriasis’/exp OR psoriasis 2. ‘eye disease’/exp OR eye disease 3. ‘uveitis’/exp OR uveitis 4. ‘Iritis’/exp OR iritis 5. ‘Iridocyclitis’/exp OR iridocylitis 6. ‘Choroiditis’/exp OR choroiditis 7. 2 OR 3 OR 4 OR 5 OR 6 8. 1 AND 7 |
